# Assessment of the Understanding of Concussion and Care Protocols Amongst Student Athletes and Coaches: A Qualitative Study

**DOI:** 10.3389/fped.2020.526986

**Published:** 2020-09-24

**Authors:** Aditya Subramaniam, Ronald Ming Ren Tan, Derrick Chan, Zhi Min Ng, Chao Yan Dong, Jasmine Xun Yi Feng, Shu-Ling Chong

**Affiliations:** ^1^Duke-NUS Medical School, Singapore, Singapore; ^2^Department of Emergency Medicine, KK Women's and Children's Hospital, Singapore, Singapore; ^3^Department of Pediatrics, Neurology Service, KK Women's and Children's Hospital, Singapore, Singapore; ^4^Education Office, Sengkang General Hospital, Singapore, Singapore; ^5^Pediatric Surgery, KK Women's and Children's Hospital, Singapore, Singapore

**Keywords:** pediatrics, head injury, rugby, football, coaches, Asia

## Abstract

**Background:** Pediatric sports-induced concussions have become a topic of interest and concern in the scientific community. Already, the literature is rich with studies that have identified numerous short-term and long-term consequences of childhood sports-induced concussions. However, there are very few studies that have identified how well the students who participate in concussion-prone sports and their coaches understand these consequences and how they can be avoided. This study aimed to explore student athletes' and their coaches' understanding of the concept of concussion and how it is managed both immediately after the injury occurs and during long-term recovery.

**Methods:** This study utilized a qualitative design. The study was conducted in local and international schools in Singapore. Participants were recruited through purposive sampling. 42 student athletes aged 13–18 who participated in rugby, softball, football, cricket, volleyball, and/or water polo were recruited. Fourteen coaches who coached these same sports were also recruited. Four focus groups and three semi-structured interviews were conducted. Data collected were then analyzed with thematic analysis. Risk factors were assessed through four domains of focus: understanding of what concussion is; attitudes toward concussion; existing protocols for treating concussion; and return-to-school and return-to-play protocols. As this is a qualitative study, outcome measures were not identified.

**Results:** Analysis of the data revealed four themes for each group. For student-athletes these included: limited understanding of concussion; non-reporting of injuries; variable supervision of athletes; and a lack of established return-to-school and return-to-play guidelines. For coaches these included: variable understanding of concussion; insufficient formal training in concussion management; limited medical support in managing injuries; and lack of understanding and adherence to return-to-school and return-to-play protocols.

**Conclusions:** Of the themes identified, the most pressing was a lack of clearly defined return-to-play guidelines. This is an urgent issue that needs to be jointly addressed by healthcare professionals and schools with evidence-based guidelines.

## Background

Sports- and recreation-related concussions (SRRC) constitute significant injuries among children and adolescents (1). An estimated 582,000–635,000 patients under the age of 19 are diagnosed with SRRC in the United States annually, and a large proportion of children and adolescents leave their SRRCs untreated (2). Serious medical complications have been found to occur after SRRCs, including post-traumatic seizures and epilepsy (3, 4). Traumatic brain injury-related neuropathological changes are potentially serious consequences of concussion in adults who play contact sports (5). Although there is a paucity of prospective studies that clearly describe the relationship between SRRCs during childhood and adolescence, and long-term neurological sequelae, there are reports that exposure to American Football before the age of 12 may be associated with an increased chance of developing impairment in executive function later in life (6).

Student athletes are known to under-report injuries for a variety of reasons, including the tendency to underestimate the severity of their symptoms (7). There is also evidence to suggest that when student athletes are treated for concussion, they are unlikely to follow medical advice and return to play prematurely, the primary reason being pressure from their teammates (8, 9). In a study by McLeod et al. (10), it was found that while most sports coaches are aware of the overt symptoms of concussion, many are unable to identify more subtle symptoms such as vision problems, sleep disturbances, and nausea. Additionally, they found that 42% of coaches felt that loss of consciousness was required for the diagnosis of concussion, despite the Consensus Statement on Concussion in Sport (11) stating the contrary. These studies show that many coaches are unaware of critical aspects of concussion and how to manage it.

Currently, there is a paucity of literature investigating the understanding of SRRCs amongst student athletes and their coaches in Singapore. There is no clear legislation and a lack of documented concussion management protocols for student athletes in Singapore. Children and athletes who are brought to primary care or tertiary institutions for concussion injuries are managed in a very heterogenous way, depending on individual physician practices. There is a lack in communication on concussion management between physicians and the community, the latter including sports coaches and students.

Through interviewing student athletes and their coaches, this qualitative study aims to explore student athletes' and their coaches' understanding of four domains of SRRC: understanding of what a concussion is, attitudes toward concussion, existing school safety protocols for treating concussion, and return-to-school and return-to-play protocols. By establishing this, we hope to be able to address the knowledge gaps on SRRCs among student athletes and their coaches and to tailor injury control education.

## Methods

We conducted focus group discussions (FGDs) for students and coaches from August 2018 to April 2019. We also conducted semi-structured interviews (SSIs) for coaches who could not attend scheduled FGDs. Both methods offered distinct advantages: FGDs promote dynamic discussion and group thinking while SSIs promote a more personalized rapport which can lead to more candid, genuine responses (12, 13).

This study was given ethical approval by the SingHealth Centralized Institutional Review Board [CIRB Ref: 2018/2746], with documentation of informed consent. All student participants gave written assent and their parents/guardians gave written informed consent. All coaches gave written informed consent in accordance with the Declaration of Helsinki.

We contacted both local and international schools in Singapore that are known for their participation in contact sports. Of six schools contacted, three schools agreed to help us recruit students and coaches. Two of these schools were part of the local system, and one was an international school. As most school children in Singapore are educated in the local system, we felt that this selection was representative of the overall student athlete population. We recruited student athletes aged 13–18 who participated in rugby, softball, football, cricket, volleyball, and/or water polo. Those with a history of prior neurological diagnoses or surgeries were not included in the study. Likewise, we recruited coaches who coached these same sports. All SSI participants were contacted individually.

This study used purposive sampling to ensure that participants who played in or coached more injury prone sports were better represented. Emphasis was placed on recruiting rugby and football players as studies have shown these sports are more prone to head injury than others (14). This would ensure that our interviews on concussion understanding and safety protocols (including return-to-school and return-to-play) were correctly targeted at the most relevant students and coaches. As this was a qualitative study, a sample size was not pre-calculated. Instead, data was gathered until a saturation point was reached where no new information emerged from the participants.

After a comprehensive literature review and input by experts (pediatricians who worked in this field and qualitative researchers), two interview guides were developed, one for students and another for coaches (Appendix in Supplementary Material). These guides were used throughout the study to ensure consistency in data collection. The interviews began with open-ended questions such as “How would you define ‘concussion'?” and were followed up with probing and clarifying questions.

All FGDs and SSIs were facilitated by three of the authors (AS, RT, and CSL), who were trained in qualitative methods. Facilitators ensured that documented informed consent was taken from all participants before the discussions began. Demographic data were collected from both students and coaches. Data on age, type of sports, and duration of participation were collected for student athletes. Data on the type of sports coached and duration of coaching were collected for coaches.

All discussions and interviews were audio recorded with a portable digital recording device and transcribed verbatim. Using the methodology outlined by Braun and Clarke (15), the data were analyzed with thematic analysis. AS, RT, and CSL familiarized themselves with the transcripts by rereading them several times and then coded each transcript using the code-recode strategy. All discrepancies in coding were resolved through discussion. This served to retain the trustworthiness of the data. Through this process, coded utterances were grouped into themes and frequency of utterances was recorded.

## Results

Four student FGDs that involved 42 student athletes were conducted, at which point data saturation was achieved. Each FGD comprised 10 to 11 participants and lasted from 55 to 65 min. Ninety-three (93%) were males and the median age of the participants was 16 years old with an Interquartile Range (IQR) of 15–16 years old. The median duration of sports participation was 5.0 years (IQR 1.0–7.0 years). Twenty (47.6%) were rugby players, 9 (21.4%) were football players, 6 (14.3%) were cricket players, 5 (11.9%) were softball players, 3 (7.1%) were water polo players, and 3 (7.1%) played other sports.

Fourteen coaches were recruited in total, at which point data saturation was achieved. 11 coaches were interviewed over two FGDs that lasted ~45–75 min, and the remaining three coaches were interviewed over three SSIs that lasted between 30 and 45 min. The median years of coaching were 14.5 years with an IQR of 8.5–21.5 years. Nine (64.2%) were rugby coaches, five (35.7%) were football coaches, and four (28.6%) were basketball coaches. One (7.1%) coached cricket, ice hockey, field hockey, volleyball, badminton, or wrestling.

Four major themes emerged from the student and coach sample data. We report the themes, along with top 2–3 most frequently discussed subthemes and supporting quotes, in Tables 1, 2, respectively (found on page 14 and 17, respectively).

**Table 1:** Students' perceptions and understanding of concussion and its management.

**Subtheme**	**Supporting quotes**
**Theme: Limited understanding of concussion**	
Identification of symptoms are based on individual experiences	“When my friend got a concussion, we found out… because he was acting a bit slow… Then, every five minutes, he would forget about what happened in the past five minutes.”
Long-term consequences are poorly understood	“I read before that if you get a series of concussions in the short term… that can get quite serious… I can't remember exactly how, I just remember there is.”
Students have a vague understanding of concussion	“…related to the nervous system or something because it affects … memory, and … disorients them.”
**Theme: Non-reporting of injuries**	
Students weigh reporting injuries against how it affects their team	“Like for example, if it's like the finals, and then you know that you feel like you're okay, you won't tell the truth… to help out the team. But if it's… just group stage or even normal training, pre-season, or post-season, you will definitely… go tell the coach or approach a medic…”
Players feel that injuries are inevitable in sport	“… injuries are really like, something that you will go through at some point… [and] not much can be done about it.”
**Theme: Variable supervision of athletes**	
Students in all sports primarily turn to coaches for injury management	“For rugby, the game can't always be stopped, like and it keeps going so, probably the first person – and this is universal I think – is the coach.”
Presence of medics or school nurse is highly variable across sports and schools	“[In football], we don't have a medic, even in our finals.”
	“In [cricket] [we have medics] only for finals or semi-final games.”
	“I would say that there have been multiple times where you don't see the nurse or the medic present on the side-lines…”
	“… usually there are some like medics on site… we're actually not allowed to play the match if the ambulance is not at the field.”
**Theme: Lack of established return-to-school and return-to-play guidelines**	
Return to school guidelines recommended by doctors are not enforced at school level	“When I had my… first and second concussions…the Singapore Rugby Union [had] a document which stated that I was suspected of a concussion… and that document was forwarded to my teachers and I had tests the following week … there were a couple of teachers who… didn't believe me… [and] didn't change the dates or anything so I still had to do the test.”
Students are allowed to return to play at the same time as they are allowed to return to school	“…there's no minimum waiting period or any tests that you actually have to do to prove that you're, you're fine or anything like that.”
Students try and get back on the field after injury as fast as possible	“… because I love both the sports I play… whether I'm injured or not, I'm really eager to get back on the pitch or back on the ice to play. Because sometimes… you have slim or not very much time to play, so you want to take as much uh, opportunities as you can to play.”

**Table 2:** Coaches' perceptions and understanding of concussion and its management.

**Subtheme**	**Supporting quotes**
**Theme: Variable understanding of concussion**	
Coaches tend to confuse signs of serious traumatic brain injury with concussion	“I think um bleeding in the nose and the mouth and the ears”
Some coaches feel loss of consciousness is required for concussion	“Another thing to is if they don't want to get up, that's another where I'm like, they're totally out…”
The nature of the impact can pre-empt coaches to think about concussion	“The nature of the incident and the impact… without even going over and assessing the child if you see how hard they were hit, you should be able to make that call….”
**Theme: Insufficient formal training in concussion management**	
Coaches recognize they are responsible for concussion management	“[Students turn to coaches because] it's the trust or the understanding that the players have after being with the coaches… we are concerned for their safety.”
Coaches learn management of concussion through first aid courses	“So it's uh, being first aid trained you will identify or learn about the signs of concussion. It comes with the experience as well. But you will not be able to help more than that. That's why you're refer to the doctor.”
**Theme: Limited medical support in managing injuries**	
Presence of medics or school nurse is highly variable across sports and schools	“For rugby, the game can't always be stopped, like and it keeps going so, probably the first person – and this is universal I think – is the coach.”
	“We used to have a nurse right at the game, but I don't know what's changed and why the nurse is not visible…”
	“… we also have, you know, 12-15 other players to look after. So we can't really take away all of our attention to that [injured] player… I mean that's why we have them there, right?”
Coaches feel medics and nurses need to be more proactive during games	“… we also have, you know, 12-15 other players to look after. So we can't really take away all of our attention to that [injured] player… I mean that's why we have them there, right?”
**Theme: Lack of understanding and adherence to return-to-school and return-to-play protocols**	
Return to school guidelines are established by doctors	“Oh, for the student who got injured, again we will actually – it depends on the doctor's prescription.”
Return-to-play guidelines exist, but are not sufficiently enforced	“Certain players look, or, diagnosed to be concussed, yeah, even by the medic and the reports written, and the next thing we see them playing the following week… But there is actually a protocol to return to play, which is two weeks, and then after that train one week… if there is a protocol then everyone should follow the protocol.”

### Students

Many students correctly identified that concussion was a form of brain injury, but struggled to identify specific symptoms. Most students could only identify specific symptoms of concussion through experiences they or their friends had gone through. Those who had experienced concussion stated that they did not properly understand what a concussion felt like until they experienced one. Understanding of concussion was greatest amongst rugby players. Long-term consequences of concussion were not well understood, however. Some student athletes felt there were no long-term consequences, while others felt the consequences were serious, but had difficulty listing any.

Almost all students felt that they should only report injuries if their injuries would hinder the team's performance. Reports of peer pressure from their own and the opposing team were shared. Students also felt that injury is inevitable in sports, and reporting every injury was impractical.

Almost all students identified their coach as the primary decision-maker in injury management, with otherwise variable medical supervision. In almost all schools, rugby players reported that medics were present for all tournament matches. Cricket and softball players shared that medics were only present at semi-final and final matches, while students playing other sports reported that medics were never on-site.

Students from all schools reported that their doctor decided when they were allowed to return to school. However, some shared instances of still having symptoms after returning to school. Most students stated they were allowed to return to play after returning to school, equating return-to-school as return-to-play. Students were extremely eager to return to play, often citing the fact that they felt pressured to participate and succeed in their sport as much as possible.

### Coaches

Many coaches demonstrated sufficient knowledge to diagnose a concussion. However, they would often confuse signs of concussion with more serious signs of traumatic brain injury (bleeding from the ears, pupil non-reactive to light, etc.). A few coaches felt that a child is not considered concussed until they lose consciousness. Many coaches agreed that the severity of impact was a reliable indicator of how likely concussion had occurred. Rugby coaches were the most familiar with concussion symptoms and signs, followed by football coaches.

All coaches agreed that they were primarily responsible for taking care of a student when they are injured. Many coaches shared that they received information about concussion management through first aid courses. No coaches had received training on management of concussion outside of these courses.

In two out of three schools, rugby coaches reported that medics were present for all matches. Football coaches shared that medics were present at quarter-, semi-, and final matches. However, in one school, coaches supervised all sports without medical assistance. Where medics were present, coaches felt that they are physically positioned too far from the pitch to witness how the injury occurred and often had to have the incident described to them, which wasted treatment time. Coaches without the support of medical supervision felt that the lack of supervision was dangerous and felt overburdened.

All coaches stated that there was no school policy on return-to-school after a concussion, and that the doctor seeing the student after their injury made this decision. As for return-to-play, most coaches allowed students to return to play once they returned to school. One school's coaches stated however that a return-to-play guideline was in place, but that not all students were following it. This guideline involved a gradual return to play that should take 3 weeks; however, it was poorly enforced.

## Discussion

We present—to our knowledge—the first study in Singapore seeking to identify the understanding of and attitudes toward concussion amongst local and international student athletes and their coaches. Students had basic knowledge of what concussion is; however, their terminology was vague and the majority lacked an understanding of the long-term consequences. Most coaches had an understanding of both the definition and symptoms of concussion, but could not differentiate signs of severe traumatic brain injury from concussion. A minority of coaches incorrectly stated that loss of consciousness was required for the diagnosis of concussion. Students and coaches agree that the coaches are primarily responsible for managing injuries; however, both groups identified a lack of medical assistance given to the coaches. Finally, the lack of adherence to return-to-play protocols was a troubling finding, and we propose that it should be addressed with urgency. An evidence-based policy that outlines how students should return to full participation after suffering from a concussion needs to be established and implemented as soon as possible.

A summary of other qualitative and cross-sectional studies that have examined concussion in other countries can be found in Table 3. Triangulating our findings with that of existing literature suggests agreement of broad themes but has also highlighted some inconsistencies.

**Table 3:** Comparison of studies exploring understanding and management of concussion.

**Research Method**	**References**	***N***	**Study Population**	**Findings**
Qualitative focus group and semi-structured interview, purposive sampling	Current Study	56	Singaporean students and coaches	Students: • Limited understanding of concussion • Non-reporting of injuries • Variable supervision of athletes • Lack of established return-to-school and return-to-play guidelines Coaches: • Variable understanding of concussion • Insufficient formal training in concussion management • Limited medical support in managing injuries • Lack of understanding and adherence to return-to-school and return-to-play protocols
Qualitative focus group, purposive sampling	Chrisman et al. (16)	50	American students	• Students are able to describe concussions with non-specific terms • Students hesitate to report concussions despite knowledge because: ◦ They wish to keep playing ◦ Non-specific symptoms could be mistaken for another etiology ◦ They felt coaches would not understand their concern
Qualitative focus group, purposive sampling	Cusimano et al. (17)	61	Canadian students, coaches, trainers, managers, game officials	• Under-reporting of concussions is very common in ice hockey • Individual motivations contribute: ◦ Students strive to win and play their role in their team effectively ◦ Coaches want to lead a winning team and help their players get into professional leagues ◦ Parents want their child to succeed in sports to improve their career prospects
Cross-sectional survey, convenience sampling	McCrea et al. (7)	1,532	American students	• Concussion often goes underreported • Common reasons include: ◦ Injury not serious enough to warrant medical attention ◦ Risk being withdrawn from competition ◦ Inability to recognize concussion
Cross-sectional survey, purposive sampling	Sye et al. (8)	477	New Zealand students	• Students were aware of concussion guidelines • Primarily received information about concussion from coaches • Only 66/477 returned-to-play after seeking medical clearance
Cross-sectional survey, convenience sampling	McLeod et al. (10)	156	American coaches	• Misconceptions about concussions exist amongst coaches • Education about identifying and managing concussions is necessary
Cross-sectional survey, convenience sampling	Lin et al. (18)	140	American coaches, athletic trainers and parents	• Knowledge gaps were identified in all groups • Coaches were the most knowledgeable, but large knowledge gaps exist

Regarding understanding of concussion in students, findings varied from study to study. McCrea et al. (7) found that students did not have sufficient understanding of either the seriousness or the symptoms of concussion. Cusimano et al. (17) found that 25–50% of all students surveyed could identify 0–1 symptoms of concussion. Chrisman et al. (16) found that, although student athletes used vague terms to describe concussion, their understanding was greater than what was reported in prior studies. Our study found that many students in Singapore have a limited understanding of how to identify a concussion injury. Educating student athletes in Singapore about signs and symptoms of concussion and giving them the vocabulary to describe their injuries may prove to be an effective way to discern between a truly minor head injury and a concussion.

Student athletes are dangerously underreporting potential concussions, but for differing reasons. The findings from McCrea et al. (7), an earlier study, suggest that this is likely due to the lack of knowledge. Covassin et al. (19) more recently demonstrated that the HEADS UP campaign by the Centers for Disease Control has increased concussion awareness amongst both students and coaches. Chrisman et al. (16), on the other hand, reported that student athletes do not report concussions despite their knowledge of symptoms and signs, instead citing three major barriers: their motivation to play their sport as much as possible, the fact that the symptoms of concussion can be attributed to a minor etiology, and fearing disapproval from coaches. Our study also showed that despite some understanding of concussion, students hesitate to report their injuries and are eager to return to play after sustaining their injury. We identified fear of disappointing their teammates as a barrier to reporting injuries. However, we did not identify fear of disapproval from coaches as a barrier for reporting injury. Instead, our data suggest that students feel comfortable reporting injuries to their coaches. Given that Chrisman et al. (16) surveyed students in America, this discrepancy may be due to cultural differences. As such, a multi-prong approach may be required. Besides increasing concussion awareness and potential consequences, encouraging open communication between student athletes, and between coaches and athletes, may help increase the chance of early diagnosis and treatment.

As for coaches, McLeod et al. (10) and Lin et al. (18) both reported that while coaches were knowledgeable, further education was necessary. McLeod et al. (10) found that, while coaches could identify overt symptoms of concussion, they could not identify more subtle signs and symptoms such as sleep disturbances and vision problems. Lin et al. (18) also found similar knowledge gaps about concussions. Both studies advocate for more specific education on concussion. Our results also showed that, while coaches could recognize overt signs and symptoms of concussions, knowledge gaps still exist that need to be addressed. Similar to these studies, we feel coaches in Singapore could benefit from more in-depth education on concussion over and above first aid courses.

Finally, our data showed that a clearly defined return-to-play protocol is urgently required. The Consensus Statement on Concussion in Sport heavily advocates for this, as does the Centers for Disease Control HEADS UP program (11, 20). Therefore, separate return-to-school and return-to-play guidelines should be clearly established. The United States Centers for Disease Control recommends a gradual, 5-step increase in activity during this period until full symptom recovery. A randomized controlled trial recently published by Leddy et al. (21) showed that this model of recovery, known as progressive subsymptom threshold exercise, could significantly reduce the duration of concussion symptoms. Distinct enforcement of return-to-play guidelines that is separate from return-to-school guidelines is a crucial step that Singapore must implement. The current standard of allowing students to return-to-play once they are back in school can be associated with adverse outcomes. A distinct enforcement of return-to-play guidelines is paramount for their safety (20).

We recognize the limitations of our study. Our purposive sampling technique limits the generalizability of the views expressed by the students and coaches to the population at large. However, we feel that purposive sampling helped to gather data from athletes who are at higher risk for SRRC and their coaches. In addition, we preferentially sampled from schools that have a higher participation in rugby in Singapore, which forced us to sample schools from higher socioeconomic backgrounds. This caused us to underrepresent the understanding of concussion among students of lower socioeconomic status. We also did not adequately sample female students who may have different experiences and viewpoints with regards to concussion. Finally, our limited age range did not account for younger athletes. The strength of this study lies in the interviews of both students and coaches, which greatly increased the breadth of our understanding of how concussion is perceived and managed. Our findings may help to generate hypotheses for future concussion research.

## Conclusions

Overall, our study showed that while students had more knowledge of concussions than previous studies reported, they struggled to identify long-term consequences. They also hesitated to report head injuries because they may be removed from the game. Coaches had a good general understanding of concussion, but may benefit from specialized training focused on concussions, and, while on-site, would prefer more skilled assistance from medics. Finally, the issue of an established return-to-play policy is an important one. The authors of this study would like to recommend that the schools in Singapore adopt a policy similar to the one recommended by the Centers of Disease Control (20). We acknowledge that this policy may be difficult to implement, but we feel this issue needs to be jointly addressed by healthcare professionals, schools, and coaches to promote the safety and well-being of student athletes with concussion.

## Data Availability Statement

The original contributions presented in the study are included in the article/Supplementary Material, further inquiries can be directed to the corresponding author/s.

## Ethics Statement

The studies involving human participants were reviewed and approved by SingHealth Centralized Institutional Review Board [CIRB Ref: 2018/2746]. Written informed consent to participate in this study was provided by the participants and their legal guardian/next of kin.

## Author Contributions

AS helped conduct the FGDs and SSIs, transcribed all FGDs and SSIs, helped analyze the transcripts, and was a major contributor in writing the manuscript. RT helped conduct the FGDs, helped analyze the transcripts, and was a major contributor in writing the manuscript. DC helped design the methodology, helped with participant recruitment, and contributed to writing the manuscript. ZN helped design the methodology and contributed to writing the manuscript. CD helped design the methodology and helped train the other authors in conducting FGDs. JF helped coordinate consent taking, data collection, and storage. S-LC helped design the methodology, conduct the FGDs, helped analyze the transcripts, and was a major contributor in writing the manuscript. All authors contributed to the article and approved the submitted version.

## Conflict of Interest

The authors declare that the research was conducted in the absence of any commercial or financial relationships that could be construed as a potential conflict of interest.

## Abbreviations

SRRC: sports and recreation-related concussion.
