# Supplementary material for: Assessment of the Understanding of Concussion and Care Protocols Amongst Student Athletes and Coaches: A Qualitative Study
Source: Front Pediatr. 2020 Sep 24;8:526986. doi: 10.3389/fped.2020.526986 (PMC7542181; doi:10.3389/fped.2020.526986)
Supplement: Supplementary file 1 [file Table_1.DOCX]

**Appendix A: Guiding Questions for Focus Group Interviews with Students**

| Domain | Guiding Questions |
| --- | --- |
| Understanding of Concussion | - What is a concussion? - What are some examples of head injuries you’ve had in your life? Do you think any of these were concussions? - What are some common signs or symptoms that indicate someone has a concussion? Are some symptoms more important than others? - What are some long-term consequences of repeated concussion? - How often in a given season do concussions occur? |
| Attitudes Towards Concussion | - Which injuries are worth reporting, and which injuries should you just try and play on with? - How does it feel when you have to sit out of a game because of an injury? - Would you be okay with missing games if you had a concussion? - How do you feel opponents view your injuries? How does it affect whether or not you report the injuries? - What are the differences in reporting an injury at the start of the season versus in the semi-finals or finals? |
| Existing Safety Protocols | - Are there any medics present, or anyone who has medical training? - What happens when someone on the field has a head injury? - Who makes the final decision on whether you keep playing or not? - At what point is a player taken to the hospital after a head injury? |
| Return-to-School and Return-to-Play Protocols | - How do concussions affect schoolwork? - What are some consequences of returning to the field too early after an injury? - What are the protocols after you get a concussion in terms of returning to school? - What are the protocols after you get a concussion in terms of returning to play? |

**Appendix B: Guiding Questions for Focus Group Discussions and Semi-Structured Interviews with Coaches**

| Domain | Guiding Questions |
| --- | --- |
| Understanding of Concussion | - What is a concussion? - Are concussions or head injuries common in the sports that you supervise? - What are some red flags that a student has a serious head injury? - What are some common signs or symptoms that indicate someone has a concussion? Are some symptoms more important than others? - If any one of your athletes had a concussion before, what happened? How could you tell? - Have you ever been exposed to education about head injuries or concussion? |
| Attitudes Towards Concussion | - Is there a difference in management between regular injuries and head injuries? - Can you explain your conversation with a student when deciding on how best to handle a head injury, be it mild or severe? - How are head injuries treated differently at the start of the season vs. during semi-finals or finals? - How much emphasis is there on techniques to avoid head injuries in practice? |
| Existing Safety Protocols | - At what point do you draw the line and force a student to sit out or seek medical attention? - Do you co-manage injuries with a medic or nurse? - How much communication is there between the coach and the medic in the event of a head injury? - Who has the final say on management in the event of a head injury? - Do you feel like the protocols established by the school can keep the students safe from concussion? What changes are necessary in your opinion? |
| Return-to-School and Return-to-Play Protocols | - How familiar are you with your school’s policy and management if one of your students has a concussion? - How long does the school require them to take time off from school? - How long are they asked to avoid contact sports? - If a student had a concussion and needed extended time to recover, what would happen to their position in the team? |
